# The interplay of cells, polymers, and vascularization in three-dimensional lung models and their applications in COVID-19 research and therapy

**DOI:** 10.1186/s13287-023-03341-4

**Published:** 2023-04-28

**Authors:** Toka A. Ahmed, Bassant Eldaly, Shadwa Eldosuky, Hoda Elkhenany, Azza M. El-Derby, Muhamed F. Elshazly, Nagwa El-Badri

**Affiliations:** 1 Center of Excellence for Stem Cells and Regenerative Medicine (CESC), Zewail City of Science and Technology, October Gardens, 6th of October City, Giza 12582 Egypt; 2 Department of Surgery, Faculty of Veterinary Medicine, Alexandria University, Alexandria, 22785 Egypt; 3 Egypt Center for Research and Regenerative Medicine (ECRRM), Cairo, Egypt

**Keywords:** COVID-19, Lung organoid, Synthetic polymers, Lung spheroid, Natural polymers

## Abstract

Millions of people have been affected ever since the emergence of the corona virus disease of 2019 (COVID-19) outbreak, leading to an urgent need for antiviral drug and vaccine development. Current experimentation on traditional two-dimensional culture (2D) fails to accurately mimic the in vivo microenvironment for the disease, while in vivo animal model testing does not faithfully replicate human COVID-19 infection. Human-based three-dimensional (3D) cell culture models such as spheroids, organoids, and organ-on-a-chip present a promising solution to these challenges. In this report, we review the recent 3D in vitro lung models used in COVID-19 infection and drug screening studies and highlight the most common types of natural and synthetic polymers used to generate 3D lung models.

## Introduction

Developing a treatment for the novel coronavirus COVID-19 has faced multiple challenges in defining the pathogenesis, target cells, the mechanisms of initiation and progression of the disease, and drug testing [1]. Development of effective COVID-19 treatment requires comprehensive understanding of the pathology caused by the new virus. As the virus’ primary target is the respiratory system and the lung tissue [2], there is a need for developing an in vitro 3D culture system that resembles the complexity of the in vivo conditions, has its own vascular support, and could be patient-specific.

Traditional 2D cultures are commonly used to study viral biology, drug discovery, and vaccine development. However, 2D culture systems do not resemble the biochemical microenvironment or the proper physiology of the human body [3]. Three-dimensional cell culture can serve as a better model for mimicking the internal microenvironment, leading to higher accuracy in the data of viral and antiviral compounds screening studies [4]. Data collected from research on scaffold-based or scaffold-free 3D culture models have shown superior accuracy and applicability to the in vivo models.

This was especially evident in studying diseases that lack a representative animal model such as brain and skin cancers [5, 6]. Moreover, 3D systems make inaccessible human tissue easier to model and investigate. For example, brain organoids were used to advance studies in developmental neurobiology and exploring brain disorders [7, 8]. In vivo studies using animal models are expensive, time-consuming, and heavily monitored in accordance with the three Rs principle, replacement, reduction, and refinement, of humane animal research [6, 9]. 3D models thus present a more relevant representation than 2D cultures and a more convenient system than in vivo models, facilitating routine experiments.

3D culture models present a valuable tool to study severe acute respiratory syndrome coronavirus 2 (SARS-CoV-2) mechanisms of infection replication and antiviral therapies. A multicellular spheroid is one of the 3D culture system methods in which cells are organized in spherical cellular aggregations [10]. In low attachment culture, spheroid formation is driven by the tendency of the cells to aggregate and self-assemble. This process is similar to what happens in nature during organogenesis [11]. 3D culture systems depend on factors that include the types of cells, the culture medium, specific growth factors, and scaffolds. In this review, we survey the components and factors that should be considered during generating lung spheroids as models for respiratory disease. These include the cell composition, matrix type, and vasculature system as well as the implementation of these models in COVID-19 studies.

### History of lung spheroid generation

Airway epithelium provides one of the first defenses against inhaled toxic substances and airborne pathogens (e.g., corona viruses) [12, 13]. Creating a reliable human lung spheroid model is necessary for studying lung development and pathologies, as well as testing new drugs or therapeutic methods to relieve respiratory diseases. Spheroids are self-assembled 3D structures where cells aggregate upon their culture on non-adhesive surfaces [14]. The first lung model having a 3D structure was generated by Benali and collaborators in 1992. In this model, human primary epithelial respiratory cells isolated from nasal polyps and tracheal gland epithelial cells were cultured on collagen matrix [15]. Generation of spheroids entailed their culture on non-adhesive culture flasks/wells [16], on hydrogel matrices or scaffolds [17], and in bioreactors [18]. Other culture methods used to mimic the physiological microenvironment include the hanging drop method (HD) [19], spinner flask technique [10], microfluidic 3D cell culture [20], and liquid overlay [21]. The hanging drop method is considered one of the simplest methods to form lung spheroids. In this method, cells are seeded into a small drop that is later inverted so that the cells aggregate at the bottom of the drop forming spheroids [19]. In a study by Liu et al., A549 adenocarcinoma human alveolar basal epithelial cell line was cultured using the HD method to form an in vitro 3D lung model [22]. This model merged a HD culture system and an air exposure system that allowed for direct contact between the cells and volatilized air toxicants, and provided a promising air exposure system that could be used in inhalational drug delivery studies and environmental risk assessment [22]. In hydrogel systems, Delgado et al. reported that seeding the cells within the gel displayed different results in spheroid structures than those on the surface of the gel [23]. In microfluidic systems, microfluidic chips can be optimized for different culture conditions to enhance lung spheroids generation [24]. Zuchowska et al. used a microfluidic lab-on-a-chip system for lung spheroid generation to test the efficiency of photodynamic therapy (PDT) on A549 spheroids. The microchip was designed to allow for direct spectrofluorometric measurements on the formed spheroids by placing the microchip in a chip holder. The study showed that PDT was lethal to cancer spheroids [25]. Culture conditions also play a pivotal role in lung cell expansion and spheroid generation. For instance, supplementing the culture medium with epidermal growth factor (EGF) and retinoic acid was reported to be crucial for lung cell growth [26]. Hagiwara et al. showed that EGF was essential for inducing branch formation of the normal human bronchial epithelial cells (NHBEs) cultured in Matrigel, as well as enhancing their migration [27]. In other studies, Rho-associated protein kinase inhibitors (ROCKi) were reported to enhance the proliferation rate of primary human airway epithelial basal cells in 2D cultures [28, 29]. To better simulate the complexity of the physiological microenvironment in the formed spheroids, other cell types such as endothelial cells (ECs) and fibroblasts were integrated [26]. Butler et al. compared tracheosphere formation derived from basal cells in two different culture media. Mitotically inactivated fibroblasts (3T3-J2) were incubated either with a ROCKi known as Y-27632 (3T3 + Y) in serum-containing epithelial growth medium, or with bronchial epithelial growth medium (BEGM) only. The study showed that tracheospheres derived from BEGM-cultured cells were dependent on the cell passage number. Passage 4 BEGM cells produced smaller tracheospheres when compared to the first passage. On the other hand, development of tracheospheres derived from 3T3 + Y cultured basal cells were not influenced by passage number [29].

When compared to 2D culture models, 3D lung spheroid models were shown to be superior in anticancer drug testing [30]. For example, TTA-A2 anticancer drug tested on A549 cell line spheroid cultured in agarose-coated plates was found to have a potent anticancer effect when compared with monolayer culture [31]. Similar data were reported in virus infection models. A549 cell line was used to generate a lung spheroid model for respiratory syncytial virus (RSV) infection using ultra-low-attachment plates. The model’s efficiency was evident in effective demonstration of RSV infection key features such as mucin secretion [32]. In another recent study, lung alveolar spheroids using A549 cells embedded in Matrigel matrix were used to validate deep learning uncovered measurement of epithelial networks (Deep-LUMEN) assay. A detection algorithm was successfully optimized to spot morphological changes in lung spheroids from bright-field images in response to different drug treatments and extracellular matrix (ECM) compositions [33]. Lung spheroids were also generated from A549 cells using rotating-wall vessel bioreactor to investigate the interaction between *Pseudomonas aeruginosa* and lung epithelial cells. In this model, *P. aeruginosa* was less effective in penetrating A549 spheroids than their monolayer counterpart and had higher levels of proinflammatory cytokines after infection, suggesting that the spheroid culture system showed more accuracy in the study of *P. aeruginosa* pathogenesis [34].

Generation of lung spheroids from type II alveolar epithelial cells (AEC2) has faced some challenges. Human-induced pluripotent stem cell-derived AEC2 were generated to overcome the difficulty of growing and maintaining human endogenous AEC2 in vitro [35]. Shiraishi et al. explored the possibility of culture expanding endogenous human AEC2 using a combination of ligands and inhibitors for crucial signaling pathways. These included Notch and fibroblast growth factor 7 (FGF7) ligands, glycogen synthase kinase 3 (GSK-3β), transforming growth factor beta (TGF-β), and bone morphogenetic protein 4 (BMP4) inhibitors. AEC2 cultured in Matrigel in transwell clear inserts successfully generated 3D spheroids [36]. Dinh et al. generated lung spheroids from whole lung tissue samples and transbronchial biopsy samples. Outgrown cells from cultured tissue explants were seeded onto ultra-low-attachment flasks generating lung spheroids, which contained a mixture of club cells, alveolar type 1 (AT1) cells, and alveolar type 2 AT2 cells together with CD90^+^ and CD105^+^ stromal-origin cells. The methodology adopted in this study could pave the way for cell-based therapies utilizing extracted lung stem cells for the treatment of lung diseases [37].

Another important type of cells used in the generation of multicellular lung spheroids is adult stem cells. Stem cell populations in the lung epithelium include basal cells [38], AEC2, pulmonary neuroendocrine cells (PNECs), and bronchioalveolar stem cells (BASCs). Basal cells spread throughout the airways and are capable of self-renewal and mucociliary differentiation into ciliated and secretory cells [38, 39]. AEC2 constitute the stem cell niche in the respiratory zone and can differentiate into alveolar epithelial type 1 (AEC1) [40]. PNECs are spread throughout the conducting airways and are capable of differentiating into ciliated cells and club cells [41]. BASCs are found between the conducting and respiratory zones border and can differentiate into AEC2 and Club cells [42, 43]. For example, Rock et al. generated 3D spheroids (bronchospheres) from primary basal cells expressing Trp-63 and nerve-growth factor receptor (NGFR). Basal cells cultured in Matrigel matrix proliferated and successfully produced bronchospheres containing a basal cell layer. A second layer of differentiated goblet and ciliated cells was also developed [38]. As mentioned, human pluripotent stem cells (hPSCs) including both embryonic stem cells and induced PSCs can be used in generating 3D airway models after step-by-step differentiation as previously reported [44–47]. However, these models generated from differentiated stem cells are composed of multiple cell types and are mostly referred to as “organoids” rather than spheroids.

### Lung spheroids and COVID-19

SARS-CoV-2 is a positive single-stranded RNA liable for the serious COVID-19. SARS COV-2 binds to the enzymatic domain of the angiotensin-converting enzyme 2 (ACE2) receptor on the surface of various cell types, including alveolar cells, intestinal epithelial cells, ECs, kidney cells, neurons, and monocytes/macrophages [48]. The spike (S) protein, which is made up of two subunits, is responsible for coronavirus binding to host cell surface receptors and membrane fusion processes (S1 and S2). After the binding of S protein to ACE2 receptor, the intracellular protease transmembrane serine protease 2 (TMPRSS2) controls the cleavage and activation of S protein in SARS-CoV-2, resulting in unlocked, fusion-catalyzed forms on the cell surface. This promotes earlier viral body entry [49, 50]. Despite the valuable data generated from 2D cultures in early SARS-CoV-2 studies, using 2D models did not accurately represent the complexity and heterogeneity of the physiological in vivo microenvironment, resulting in inaccurate outcomes in drug screening [3]. 3D cultures represent a promising alternative model that accurately mimic the complexity of the physiology and microenvironment of the organ where the infection of SARS-CoV-2 naturally occurs [4]. Table 1 provides a summary of the studies that used 3D culture models, composed of organoids or spheroids to mimic the physiological environment of human organs in cases of SARS-CoV-2 infection (Table 1). Many of these models were shown to be superior to traditional culture for drug discovery. Lung organoid models used in SARS-CoV-2 research are based on the development of the distal airway, including AT2 cells, and express high levels of ACE2 and TMPRSS2 which are required to study the viral infection and pathogenesis [51–53].

**Table 1:** Three-dimensional lung models used in COVID-19 studies

3D lung model	Cell composition	3D matrix/method	Application of the model	Key findings	References
Lung organoid	hPSCs	100% Matrigel	Screening compounds for SARS-CoV-2 inhibition	Susceptibility of (hPSC-LOs) specifically AT2s to SARS-CoV-2 infection Similar chemokine induction pattern to COVID-19 patients	[58]
Distal lung organoids	Alveolar epithelial type II (AT2) or KRT5^+^ basal cells	Basement membrane extract II	Developing SARS-CoV-2 infection model	Successful infection of AT2, basal cells Identifying SCGB1A1^+^ club cells as a target population No infection of ciliated cells	[59]
Alveolar spheroids	hAT2	Matrix-free model	Developing SARS-CoV-2 model	Rapid viral replication and high expression of proinflammatory and interferon-associated genes in hAT2 cells	[52]
Alveospheres	Human AT2 cells/ pneumocytes	Matrigel	Developing culture method for enhanced propagation and differentiation of alveospheres and test their validity for SARS-CoV-2 studies	40% of AT2s expressed ACE2 and about 80% were positive for TMPRSS2 activation of type II IFN pathway in AT2s Pre-treatment with IFNs shows prophylactic effectiveness in alveolospheres	[60]
(hBO)	Human bronchial epithelial cells	Matrigel—GFR basement membrane matrix	Establishing a proximal lung model for SARS-CoV-2 drug screening	High expression of ACE2 and TMPRSS2 after SARS-CoV-2 infection of hBO Increased levels of cytotoxicity, intracellular viral genome, progeny virus, and moderate elevation in type I interferon signal	[61]
Lung organoid	Human lung airway epithelial cells, basal stem cells, and pulmonary Microvascular ECs	Microfluidics Organ-on-a-chip + ALI	Developing organ-on-a-chip model to study influenza and SARS-CoV-2 viruses	Differentiated airway cells at ALI on-chip showed large increases in protein and mRNA expression levels of the SARS-CoV-2 receptors (ACE + TMPRSS) Two out of seven clinically approved drugs showed significant entry inhibition of the pseudotyped SARS-CoV-2 virus	[63]
hAWOs + hALOs	(hESCs)	Matrigel	Developing a model for SARS-CoV-2 infection and drug testing	All viral infected cells expressed ACE2 but not all ACE2-expressing cells were infected Ubiquitous expression of TMPRSS2 in both hAWOs and hALOs Club cells were identified as new target cells of SARS-CoV-2 Significant down-regulation in metabolic processes especially lipid metabolism after infection	[66]
Complete lung organoid	Lung cells obtained from biopsies + hiPSC-derived alveolar type-II (AT2) pneumocytes	Cold Matrigel	Producing a scalable and cost-effective 3D complete lung model	Infected ALO monolayers had the best recapitulation to transcriptomic signatures in COVID-19 patients Distal alveolar differentiation (AT2 → AT1) 3D-to-2D conversion virtually abolished the AT2 signatures, showing prominent emergence of AT1 signatures	[64]
Alveolar organoids	Primary epithelial cells isolated from normal human distal lung tissue	Cold Matrigel	Establishing 3D model to study the response of proximal and distal lung epithelium to SARS-CoV-2 infection	Transcript profiles of infected organoids showed high levels of SARS-CoV-2 viral RNA High levels of cytokines and antiviral response genes No significant change in the expression of AT2 cell marker, as ACE2 and TMPRSS2 was observed	[67]
Lung organoids	hESC	Matrigel	Investigating the high prevalence of severe complications of SARS-CoV-2 in men and relative immunity in children	Androgen increased the expression of viral receptors and SARS-CoV-2 infectivity Screened antiandrogenic drugs decreased SARS-CoV-2 infectivity	[68]

SARS-CoV-2 does not only cause lung damage, but also affect several organs such as the liver [54], the kidneys [55], the cardiovascular system [56], and others that express ACE2. However, the highest expression of ACE2 occurs in human type II alveolar cells in the lungs, which is the most affected organ in Covid-19 patients [57]. Generation of lung organoids has been recently pursued as one of the most relevant models for SARS-CoV-2 studies. For instance, Han et al. succeeded in developing a lung organoid model from hPSCs (hPSC-LOs) using 100% Matrigel dome matrix. HPSC-LOs, specifically the alveolar type-II-like cells, were susceptible to SARS-CoV-2 infection and induced chemokines in a pattern that was similar to that of COVID-19 patients [58]. On the other hand, Salahudeen et al. used basement membrane extract II (Trevigen) matrix to develop a model of distal lung organoids derived from human AEC2, or KRT5^+^ basal cells. To facilitate the viral access to the ACE2-expressing luminal cells, the researchers used an apical suspension culture to allow the virus easy access to the intact apical surface and to reach the site of infection. AT2 cells, basal cells, and SCGB1A1^+^ club cells were identified as a target for viral infection, while no infection was observed in ciliated cells. The researchers concluded that alternative culture conditions may be required [59].

Alveolar spheroids represent another model for SARS-CoV-2 studies. Youk et al. established a matrix-free model of 3D human alveolar stem cells (hAT2) derived from primary human lung tissue using chemically defined culture conditions. The aim of this model was to stimulate self-organization of single hAT2 cells into alveolar-like 3D structures. Rapid viral replication was achieved, and high expression of proinflammatory and interferon-associated genes in hAT2 cells was observed, demonstrating a robust endogenous innate immune response [52]. Similarly, Katsura et al. developed a culture system for enhanced propagation and differentiation of alveospheres, derived from human AT2 cells/ pneumocytes. Culture conditions were optimized using murine and human AT2s on Matrigel-coated plates in a serum-free feeder-free medium. Results showed the activation of type-II IFN pathway in AT2 cells. Furthermore, pre-treatment with interferons (IFNs) showed prophylactic effectiveness of the alveolospheres [60].

To establish a proximal lung model, Suzuki et al. used cryopreserved human bronchial epithelial cells to develop human bronchial organoids (hBO) on Matrigel growth factor reduced (GFR) basement membrane matrix. The study showed high expression of ACE2 and TMPRSS2 after infection of hBO with SARS-CoV-2. In addition, increased cytotoxicity, intracellular viral genome, progeny virus, and moderate elevation in type I interferon signal were observed [61].

Using microfluidics technology [62], Si et al. used air–liquid interface (ALI) to generate 3D lung models to mimic surfactant-dependent alveolar homeostasis [63]. Human lung airway epithelial basal stem cells and pulmonary microvascular ECs were tested on pseudotyped SARS-CoV-2 virus on a microchip. Differentiated airway cells at the ALI showed large increases in protein and mRNA expression levels of both SARS-CoV-2 receptors ACE2 and TMPRSS. Despite these interesting findings, only two out of seven clinically approved drugs, amodiaquine and toremifene, showed significant entry inhibition of the pseudotyped SARS-CoV-2 virus [63].

Using the same ALI method, Tindle et al. used cold Matrigel matrix to establish adult lung organoids (ALOs) complete with both proximal airway and distal alveolar epithelia [64]. This method showed both cost-effectiveness and scalable 3D complete lung model. When comparing the infection of ALOs with human-induced pluripotent stem cells (hiPSCs)-derived AT2 pneumocytes, the first showed the best recapitulation to transcriptomic signatures in COVID-19 patients, whereas distal alveolar differentiation (AT2 → AT1) was crucial for producing a profound host immune response [64].

To enhance the generation of 3D organoids, Pei et al. developed two 3D models of human airway organoids (hAWOs) and alveolar organoids (hALOs). Both organoids were derived from human embryonic stem cells (hESCs) using Matrigel along with defined growth factors. All viral infected cells expressed ACE2 but not all ACE2-expressing cells were infected. TMPRSS2 was expressed ubiquitously in both the hAWOs and hALOs, in contrast to the restricted expression pattern of ACE2. In addition to club cells, a type of bronchial epithelial cells was identified as new target cells of SARS-CoV-2. Furthermore, significant down-regulation in metabolic processes especially lipid metabolism after SARS-CoV-2 infection was reported [65, 66].

Mulay et al. used an ALI culture system on Matrigel to create a primary human lung epithelial 3D lung model to study the response of proximal and distal lung epithelium to SARS-CoV-2 infection. Transcript profiles of infected organoids showed high levels of viral RNA, indicating successful replication and gene transcription in the produced organoids, in addition to high levels of cytokines and antiviral response genes. However, no significant change in the expression of AT2 cell markers such as ACE2 and TMPRSS2 was observed [67].

To investigate the high prevalence of severe complications of SARS-CoV-2 in men and relative immunity in children, Samuel et al. used lung organoids derived from hESCs using Matrigel matrix. Androgen was shown to increase the expression of viral receptors, leading to increased SARS-CoV-2 infectivity, and antiandrogenic drugs were shown to decrease the infectivity [68].

### Vascularization of 3D models

The vascular network formation is the result of the orchestration between the vasculogenesis and angiogenesis [69]. Angiogenesis is the formation of new blood vessels from existing ones; it comprises ECs proliferation, migration, polarization, sprouting, maturation, and stabilization to end with new functional blood vessel [70, 71]. On the other hand, vasculogenesis is the de novo formation of the blood vessels through the differentiation of mesodermal-derived hemangioblasts. Angioblasts are the ECs precursors that develop into intraembryonic vasculature of the neural tube, limbs, and organ-specific vascular plexus [72, 73]. In the early stages of the embryonic development, embryonic stem cells (ESCs) give rise to three germ layers, “ectoderm, endoderm, and mesoderm.” Both vascular ECs and hematopoietic cells have mesodermal origin [74]. Vasculogenesis in the embryo is followed by angiogenesis which in turn comprises endothelial cell division and sprouting migration, controlled by the vascular endothelial growth factor (VEGF)-Notch pathway [75, 76]. The newly formed blood vessels become lined internally with stalk cells, followed by specialization into arteries and veins, under the influence of several internal and external signals including ephrinB2-EphB4, VEGF, Notch, delta-like ligand 4 (Dll4), and Chicken Ovalbumin Upstream Promoter Transcription Factor II [77–79]. Sporulation is followed by elongation and maturation of the newly formed blood vessels, aided by the contractile mural cells [80]. Mural cells play important role in angiogenesis via matrix metalloproteinase (MMP) secretion [81], basement membrane formation, and ECs permeability regulation [82, 83]. The absence of vascularization in the engineered tissues leads to cell starvation and hypoxia which in turn ceases the cell growth and may lead to alteration in the cell phenotype and loss of functionality, and eventually cell death [84, 85]. The absence of vascularization also has a significant role in hindering the tissue’s regenerative capacity [86, 87]. Vascularization could be introduced to the engineered spheroids or organoids to mimic the original physiological or pathological state. Despite the plethora of organoid models for drug screening and disease modeling, most of these are not complete due to the absence of vasculature [88]. The pre-vascularized organoid is an excellent platform to study the role of angiogenesis in organogenesis and regeneration [89, 90]. Vascularization can be introduced to the organoid either by the induction of angiogenesis or incorporation of microvasculature network [91]. ECs are the most common cells to be incorporated for vascularization, due to their inherent angiogenic properties and their ability for self-assembly into a vascular network that integrates with host vasculature [92]. However, the limited proliferation potential of the terminally differentiated cells and the absence of the supporting mural cells represent a technical challenge [93, 94]. One of the important vascularization approaches is ECs spheroid culture which was first developed to mimic the in vivo cell–cell interaction [95]. Spheroid culture suffers the development of a hypoxic core that results from cell aggregation and the significant decrease in oxygen diffusion. The hypoxic core thus stimulates the expression of hypoxia-inducible factor-1 (HIF-1) and its downstream target VEGF which in turn binds to VEGF receptor and modulates the angiogenesis through extracellular regulating kinase 1/2 (ERK-1/2) and mitogen-activated protein kinase (MEK-1/2) pathways. These events shift the environment to a proangiogenic one that better stimulates physiological angiogenesis. However, vascular network should contain other components than the endothelial tube, such as smooth muscle cells, basal lamina and pericytes (PCs) [96]. Indeed, pre-vascularized, spheroids that comprise both vascular cells such as human umbilical vein endothelial cells (HUVEC) and other types of cells showed an obvious ability to mimic the complexity of tissue microenvironment in cases of cardiac tissue [97] and hepatic tissue [98]. Moreover, HUVEC were reported to be used in combination with human umbilical artery smooth muscle cells (HUASC) to generate a vascularized porcine intestinal model [99]. In another study, a spheroid model comprising HUVEC and HUASC showed a decline in the level of platelet-derived growth factor beta (PDGF-B) compared with HUVEC alone. This may be attributed to the potential of the spheroid model to mimic the in vivo environment, where PDGF-B disappears after capillary maturation [100]. Cardiac spheres that comprise both HUVEC and cardiomyocytes displayed capillary-like network formation that contributed to the viability and maintenance of the functionality, phenotype, and longevity of the cardiomyocytes [101]. Interestingly, macrophages were reported as another supporting type of cells that can contribute to remodeling of the microvasculature during angiogenesis via their growth factor-rich secretome, phagocytosis, trafficking, and proliferation behavior. Dohle et al. reported that co-culture of ECs and primary osteoblasts exhibited an increasing number of capillary-like structures when exposed to macrophages compared to controls. This could be attributed to the elevated levels of VEGF in the co-culture condition [102]. Walser et al. demonstrated that co-culture of spheroids of human osteoblasts and dermal endothelial cell (DECs) successfully formed microvascular-like capillaries that interconnected with host vasculature when transplanted in vivo. Human dermal fibroblasts (HDFs) co-culture exerted no significant effect on the vascularization level in that model [103]. Mesodermal progenitor cells (MPCs) derived from human iPSCs have been used to generate vascularized organoids overcoming the limitations of the ESCs. Wörsdörfer and colleagues generated vascularized neural and tumor organoids by co-culture of MPCs with the neural spheres and tumor cells respectively. The in vitro formed blood vessels displayed the potential to connect the blood vessels in the chicken chorion allantois membrane (CAM) [88]. In contrast to using terminally differentiated ECs, this model showed hierarchic organization of the blood vessels and assembly of mural cells into the vessel wall. Moreover, high plasticity and maturation in the endothelial network of the growing organoids has been reported, including responsiveness to the proangiogenic and anti-angiogenic factors.

**Endothelial progenitor cells (EPCs),** first reported by Asahara et al. as a circulating CD34^+^ population in the peripheral blood, are considered an important cell source for vascularization. EPCs were shown to integrate with the blood vessels and restore neovascularization after injection into the ischemic hind limb of a mouse model [104]. Loibl et al. reported that co-culture of EPCs and mesenchymal stromal cells led to differentiation of the latter into PC-like cells expressing the PC markers Neuron-glial antigen 2 (NG2) and alpha smooth muscle actin (α-SMA) [105]. Moreover, using polyurethane scaffold, Duttenhoefer et al. successfully developed a vascularized construct enriched with CD34^+^ and CD133^+^ EPCs and mesenchymal stromal cells** (**MSCs) [106].

**MSCs** are another important cell source for vascularization of engineered tissues, by means of differentiating into the endothelial-like cell under the influence of a pre-defined differentiation cocktail. Wang et al. reported that treatment of bone marrow-derived MSCs with VEGF and basic fibroblast growth factor (bFGF) for one week induced the expression of EC markers [107]. Au et al. reported that co-culture of bone marrow-derived MSCs and HUVECs induced the differentiation in the former into functioning pre-vesicular cell [108]. The same results were reported by Laranjeira et al. who showed that co-culture of human DECs and MSCs caused the latter to express high levels of collagen 1 and VEGF165 [109], and elevated the levels of their angiogenic secretome [110]. Stenderup and colleagues demonstrated that when ECs were co-cultured with MSCs, each cell type migrated toward its specific niche and formed separate aggregates that contacted sporadically. The culture medium was found to greatly influence the differentiation potential and proliferation of the co-cultured MSCs toward ECs [111, 112]. Co-cultured MSCs with ECs in fibronectin-containing collagen contributed to the vasculogenesis of the engineered construct [86]. In another study by Deegan and colleagues, dynamic culture conditions had a positive effect on cellular functions, arrangement, and interaction between the HUVEC and MSCs [113]. Using a suitable cocktail of growth factors enhanced cell differentiation in generating vascularize organoids. A study by Morita et al. showed that human fibroblasts had the potential to be converted into functional ECs using erythroblast transformation-specific transcription factor 2 (ETV2) [114]. ETV2 contributed a significant role in generating endothelial lineages from HDFs with more than 90% efficiency through delivery of modified mRNA encoding ETV2 [115]. More recently, Cakir et al. have incorporated 20% of doxycycline-inducible ETV2 gene-transduced iPSCs to the 3D aggregates during their initial formation. Their data showed successful formation of vascular structures within the cerebral organoids [116]. Although many studies showed that vascularization is an important factor to generate biomimetic 3D models of numerous tissues, only few studies reported the generation of vascularized lung spheroids or organoids. Further studies to enhance the generation of vascularized 3D lung models are needed to accelerate their application in drug testing and molecular studies for COVID-19 research.

### Polymers for 3D cell culture and spheroid formation

**Fig. 1:**
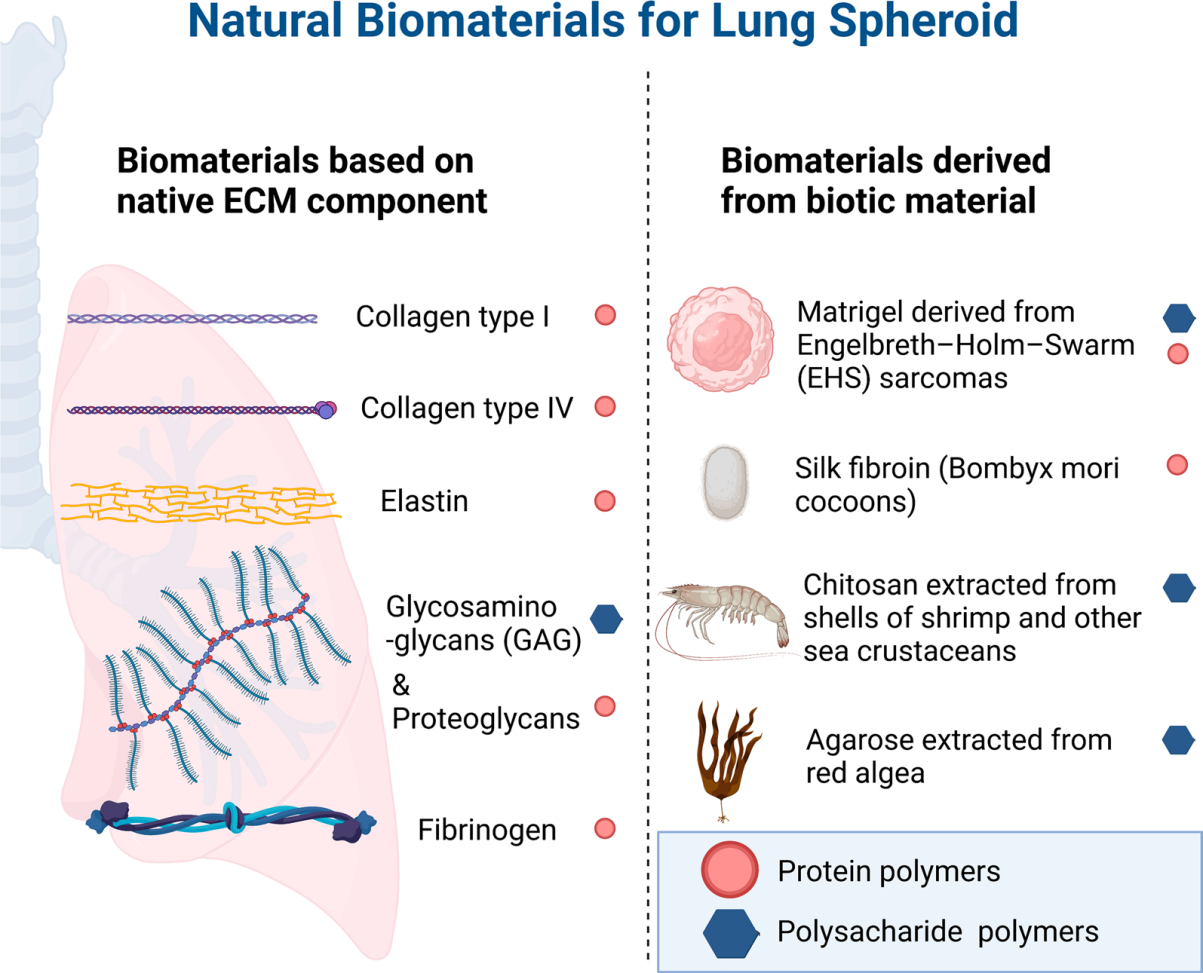
Schematic diagram showing the natural biomaterials for lung spheroid generation. This figure was created with BioRender.com

Scaffolds are considered the basis of tissue engineering and essential for organ reconstruction [117]. The generation of well-patterned and functional organoids depends on the ECM structure and mechanical properties of the biomaterial used [118]. Natural and synthetic biomaterials have been utilized reliably to generate organoids with physicochemical and biomechanical properties that mimic the native tissues and reliable for disease modeling and drug discovery. Natural biomaterials represented in protein and polysaccharide polymers have been investigated in various tissue engineering applications (Table 2, Fig. 1). Herein, we review the properties of the biomaterial as a suitable matrix for lung tissue regeneration.

**Table 2:** Properties of natural and synthetic polymers exploited for culture of 3D cellular spheroids

Type	Name	Features to support spheroid culture	References
Protein polymers	Collagen	Biocompatibility No cytotoxicity Good tensile strength Low immunogenicity Lack mechanical strength and structural stability upon hydration	[122, 215]
	Silk fibroin	Adhesive properties High mechanical strength Biocompatibility	[129, 131]
	Fibrin	High availability and biocompatibility viscoelastic properties Low cost	[135, 216]
	Matrigel	Liquid at temperatures below 8 °C Solid at temperatures above 8 °C Biocompatibility Mechanical properties Heterogenic variability from batch to batch	[217–219]
Polysaccharide polymers	Glycosaminoglycans	Biocompatibility Maintain viscoelasticity of tissue	[164]
	Hyaluronic acid	Hydrophilicity Biocompatibility Limited immunogenicity	[169, 220]
	*Chitosan*		
	Shrimp chitosan	Elongated pores Low water absorption properties and degradation rate Enhanced cell attachment	[172, 179, 221, 222]
	Fungal chitosan	Polygonal pores High water absorption and degradation properties Enhanced cell attachment and proliferation	
Synthetic polymers	PNIPAm	Thermoresponsive properties Biocompatibility	[3, 20, 202, 204]
	PDMS	Hydrophobicity Biocompatibility High oxygen permeability Simple preparation techniques	[20, 210, 211]
	PCL	Rheological and viscoelastic properties (i.e., solubility and low boiling point) accommodating different fabrication techniques Low cost	[2, 19, 217, 218]

## Natural biomaterials

### Protein polymers

#### Collagen

Collagen is a natural polymer and one constituent of the lung ECM. Collagen forms about 15% of the dry weight of human lung [119], and can be identified in alveolar interstitium, and bronchi and blood vessels in several types (Type I, III, IV, V, and VI) [119, 120]. Collagen possess high tensile strength by which it can control parenchymal expansion and prevent airway collapse [121]. Hamilton, et al. [122] reported that collagen type I scaffold protected tracheospheres generated from airway lung basal cells and fibroblasts, and enhanced vascularization upon implantation into decellularized tracheal scaffold. In addition to being biocompatible, collagen was found to be non-cytotoxic, reduced the fibrinogenic phenotype of bone marrow mesenchymal stem cells (BM-MSCs), and enhanced their regenerative capacities [123]. Collagen type I loaded with BM-MSCs generated cartilage-like tissue similar to native ECM, both mechanically and morphologically [124]. Collagen has been shown to trigger MMP-dependent directional migration of stromal cells from endometrial spheroids [125]. Type I collagen mixed with glycosaminoglycan (GAG) was found to maximize its potential to form matrix for better alveolar regeneration [126]. The alveolar-like structures generated by this scaffold had the ability to contract, as confirmed by positive expression of α-smooth muscle actin. Type I collagen also provided a suitable matrix for generation of multicellular tumor spheroids to study lung cancer [127].

#### Silk fibroin

Silk fibroin (SF) is a natural protein polymer derived from *Bombyx mori cocoons*. SF has been classified as an excellent biomaterial for tissue regeneration [128]. One of its components is sericin, which was classified as an adhesive protein [129], characterized by a controllable degradation rate [130]. Silk fibers showed high mechanical strength in which 7.25 MPa estimated the ultimate tensile strength. These properties prompted its application in trachea tissue regeneration [131]. Three-dimensional SF scaffold loaded with tracheobronchial epithelial cells enhanced epithelial regeneration with less fibrosis in experiments to reconstruct a tracheal defect in rabbit [131]. Electrospun scaffold of SF could also provide ECM that is architecturally and morphologically similar to native tissue [132]. Human umbilical cord MSCs (UC-MSCs)-loaded SF scaffold enhanced cell proliferation, reepithelization, vasculogenesis and most importantly reduced scar formation in skin regeneration [132]. SF scaffold loaded with human adipose stem cells (ASCs) also have shown strong angiogenic potential using chorioallantoic membrane model [133].

#### Fibrin

Fibrin biopolymer formed after activation of fibrinogen by thrombin plays role in blood clotting, inflammatory response, and wound healing [134]. The availability, porosity, biocompatibility and low cost of fibrin hydrogel present it as an acceptable scaffold for tissue engineering [135]. Fibrin-based scaffolds are characterized by their ability to maintain their architecture, while lacking contractile properties. These properties supported their applications in particular tissue scaffolds thanks to their high reproducibility and integrity compared to collagen-based matrix [136, 137]. For example, cross-linking fibrin hydrogel with alginate–chitosan composite enhanced the cell differentiation and generation of self-organized liver organoids [138]. Combining fibrin with polypropylene fumarate enhanced the vascularization of neobone tissue [139]. In combination with laminin, fibrin scaffolds have been demonstrated to provide physical support for organoid formation and long-term expansion [140]. Moreover, fibrin hydrogel mixed with other materials, such as polyethylene glycol (PEG) was used to tailor a 3D lung adenocarcinoma model [141], while a scaffold of fibrin and collagen microfibers was successfully used to investigate the vascular invasion of cancer [142]. In order to modulate the poor mechanical properties of fibrin, agarose has been used to increase its stiffness [143].

#### Matrigel

Matrigel is a hydrogel extracted from murine Engelbreth–Holm–Swarm sarcomas [144]. Matrigel consists of a diverse mixture of ECM proteins (laminin, collagen IV, and enactin), proteoglycan, and growth factors (including bFGF, EGF, insulin-like growth factor-1 (IGF-1), TGF-β, PDGF, and NGF) [145–147]. The high versatility of Matrigel to enhance 3D culture has been reported in different studies. For instance, Matrigel was used for culturing iPSCs in 3D culture to investigate the endochondral pathway [148], cerebral organoid formation [149], liver [150, 151], kidney [152], and intestinal crypt organoid [153]. However, some drawbacks have been reported including batch-to-batch variability and hard to determine factors that contributed to the regeneration due to composition complexity [154, 155]. Using Matrigel in microfluidic system has provided an optimal environment to enhance cell migration at low concentration, but not at high concentration, and this was attributed to excessive cellular attachment [156]. It is worth mentioning that Matrigel has been used intensively in spheroid generation. Matrigel was the matrix of choice to study the complex differentiation process of iPSCs into ventral–anterior foregut spheroids and ultimately generation of lung organoids [157]. Human lung spheroids generated on Matrigel were used to investigate the lung’s regenerative potential of pulmonary fibrosis in rodents [158]. In this study, it has been demonstrated that lung spheroid cells (LSCs) encapsulated into Matrigel could successfully differentiate into alveolar-like cells and generate alveoli-like structures that displayed positive aquaporin-5 (lung epithelial cell marker) staining. Matrigel has been successfully used to form primary lung tumor spheroids for testing antitumor drugs and understanding the tumor’s pathophysiology [159–161].

### Polysaccharide polymers

#### Glycosaminoglycans (GAGs)

GAGs are long, linear, and heterogeneous polysaccharides located at the alveolar interstitium, sub-epithelial tissue, and bronchial wall [162]. GAGs play an important role in the lung viscoelasticity [163]. Recently, it has been demonstrated that losing GAGs during lung decellularization had negative impact on cell attachment and growth, requiring replating the GAGs for better tissue regeneration [164]. However, combination of GAGs with collagen type I showed effective alveolar regeneration [126]. It has been reported that GAGs were involved in RSV infection [165]. Interestingly not all the GAGs had contribution to viral infection but only GAGs containing iduronic acid (like heparan sulfate and chondroitin sulfate B). Treating cells with protamine sulfate or GAG-destroying enzyme was shown to reduce viral infection [165].

In another study, chondroitin sulfate, a major population of GAGs, enhanced Japanese encephalitis viral replication in a neural cell line and the brain [166]. GAGs-related scaffolds may be used for SARS-CoV-2 disease modeling in vitro. We have recently reported that SARS-CoV-2 spike glycoprotein may interact with GAGs of the host cell surface as a mechanism to facilitate its entry to the host cell [167].

#### Hyaluronic acid

Hyaluronic acid (HA) is non-sulfated glycosaminoglycan that resides in all native tissue ECM and body fluids [168]. HA has shown a potential effect to stimulate cell proliferation, migration and attachment by interaction with CD44, receptor for hyaluronan-mediated motility (RHAMM), and intercellular adhesion molecule 1 (ICAM-1) receptors [169]. HA was also found to promote angiogenesis through RHAMM-transforming growth factor beta receptor-1 (TGFβRI) signaling pathway [170]. Interestingly, HA has shown a protective and therapeutic effect on lung tissue and alleviation of the toxic effect of bacterial pneumonia in an ex vivo model of lung injury [171]. On the other hand, HA possesses poor mechanical properties, which were overcome by cross-linking using chemicals [172], for example, mixing with collagen improved HA mechanical strength [173] It has been demonstrated that HA significantly increased stemness as confirmed by upregulation of NANOG, octamer-binding transcription factor 3/4 (OCT3/4), sex-determining region Y HMG-box 2 (SOX-2), and stage-specific embryonic antigen 3 (SSEA-3) gene expression and cell survival of ASCs compared to monolayer cultures [174]. In cosmetic applications, the viscoelastic properties of HA hydrogel supported its application in augmentation therapy for wrinkles [175]. Injection of human MSC spheroids encapsulated in HA hydrogel was effective in the regeneration of the fibrotic esophagus [176]. Moreover, hyaluronan–chitosan (HA–CS) biomaterial was used to generate a 3D lung tumor spheroid to investigate the cross talk of cancer cells and MSCs. Co-culture of MSCs and cancer cells on HA–CS was found to generate tumor spheroid in which MSCs aggregate in the spheroid center; however, the cancer cells aligned at the outer surface known as core–shell structure. The size of the spheroid was reported to correlate to the initial seeding cell number. Moreover, HA–CS scaffold was reported to increase cancer cells stemness, migration, and tumorgenicity [177].

#### Chitosan

Chitosan is a natural polysaccharide produced by deacetylation of chitin, derived from shells of shrimp and other crustaceans [178]. Chitosan extracted from *Gongronella butleri* fungi was reported to have superior biological and mechanical properties to shrimp chitosan [179]. In order to produce 3D porous scaffold, thermally induced phase separation (TIPS) technique are used to produce pore sizes that range from 1 to 250 μm depending on temperature and water content [180]. Increasing the porosity of chitosan was found to lead to increasing the surface area and decrease the scaffold elasticity; hence, integration with other polymers such as collagen was recommended to maintain the elasticity [181]. One of the chitosan disadvantages is low mechanical resistance and stiffness [181]. This can be optimized by cross-linking with other materials such as polyethylene glycol and starch [182]. Adding chitosan to collagen scaffold for skin regeneration was shown to enhance the biocompatibility and biostability of the scaffold [181]. This combination was successfully used to generate 3D lung model to investigate influenza A infection [183]. In this study, collagen–chitosan 3D scaffold was shown to enhance the viability of the primary human small airway epithelial cells (HSAECs), and preserve the morphological characteristics of the native lower airway. 3D cultured HSAECs could successfully mimic the same in vivo airway epithelium characteristics as confirmed by aquaporin-5 and cytokeratin-14 expression [183].

#### Synthetic polymers

Synthetic polymers have been extensively used as supporting matrices for 3D culture of cellular spheroids for drug delivery and screening [184]. They offer several advantages over natural polymers thanks to their highly tunable properties that facilitate altering and optimizing experimental parameters to mimic the mechanical properties of various tissues in the in vivo environment [185]. However, since synthetic polymers are usually biologically inert, functionalization with cell adhesion peptide domains (e.g., arginylglycylaspartic acid (RGD)) is essential to promote cell adhesion and spheroid formation [186]. The tunable mechanical properties of synthetic polymers, depending on fabrication conditions, cross-linking, and copolymerization with natural and synthetic agents, allow for their exploitation in different biological applications. PEG is a hydrophilic polymer used frequently for the preparation of hydrogels. It exhibits relatively good mechanical properties. In addition to non-toxicity, PEG does not elicit an immune response, which makes it highly biocompatible and suitable for many biomedical applications. The properties of PEG are generally tunable with different fabrication methods including chemical and physical polymer cross-linking [187–189].

As a synthetic polymer, it lacks the biological moieties necessary for cellular activity, which makes it more suitable for ECM components during hydrogel preparation. Hybrid hydrogels incorporating gelatin within the photo-cross-linked PEG network improved cell adhesion and mechanical properties [188]. Moreover, the mechanical properties of PEG can be altered to match tissue stiffness that influences cell behavior. Through introducing a soluble allyl-presenting monomer to PEG–diacrylate hydrogel precursor solution before cross-linking, it was possible to reduce the stiffness within soft tissue regime (e.g., neural tissue) from 5.1 ± 0.48 kPa to 0.32 ± 0.09 kPa which demonstrates the potential of the compliant hydrogel system for examining the cell behavior in soft tissues [190]. PEG-based hydrogels were fine-tuned for lung spheroid generation. For instance, Gill et al. used a PEG hydrogel functionalized with two different bioactive peptides and mixed with murine lung adenocarcinoma cell line 344SQ exhibiting an expression behavior similar to human non-small cell lung adenocarcinoma. Arginine–glycine–aspartate–serine peptide (RGDS) was used for cell adhesion, while a MMP-sensitive peptide, GGGPQGIWGQGK (PQ), was used as a cell-degradable hydrogel backbone that can be cleaved by matrix metalloproteinase-2 (MMP-2) and matrix metalloproteinase-9 (MMP-9). Matrix stiffness was optimized by varying percentages of PEG in the matrix. This system successfully generated lumenized epithelial spheres similar to those observed in Matrigel culture. These experiments provided key evidence for the built-in differentiation capacity of cancer cells independent of matrix components. Most importantly, this study provided a reliable and modifiable tool to investigate the influence of ECM on tumor behavior by integrating other bioactive peptides and ECM ligands in the hydrogel system [191]. In another study by Roudsari and colleagues, the same PEG-based hydrogel system was modified by creating a dual layer hydrogel system where lung cancer cells (344SQ) are localized in one layer and HUVECin the other one. This study reported the development of a lung tumor angiogenesis model that could be used to investigate the interactions between vascular and cancer cells as well as the influence of vascularization on tumor progression [192].

#### Poly(N-isopropylacrylamide) (PNIPAm)

Poly-N-isopropylacrylamide (PNIPAm) is a smart thermoresponsive polymer used extensively in cell culture applications. Mediated PNIPAm substrates provide suitable conditions for cell adhesion and growth under physiological conditions. Additionally, the thermoresponsive properties of PNIPAm as a hydrophilic compound that becomes swollen at temperatures below 35 °C, and a hydrophobic one that shrinks at temperatures above 35 °C, allow efficient cell isolation. This can be achieved through altering surface hydrophobicity by means of changing temperature without the need for trypsin or other chemical agents [193–195]. Microgel particle size in the PNIPAm hydrogel was optimized and used for multicellular spheroid generation. This achieved reduced cellular uptake and improved physical properties (i.e., reduced shrinkage) [196, 197]. The biocompatibility of PNIPAm was validated by Capella and colleagues, who reported that PNIPAm hydrogel exhibited good biocompatibility, favorable cell attachment, and non-cytotoxic effect on A549 cell lines. These favorable biocompatible effects proved effectiveness in lung spheroids generation without detectable DNA damage [198]. In another line of research, PNIPAm-based hydrogel was also used for drug screening on human lung fibroblast (HLF) spheroids [195], and formation of ASCs spheroids [199]. Dhamecha et al. reported the use of PNIPAm-based hydrogel microwell array (PHMA) for the high-throughput generation of spheroids using different cell lines including HLF and A549. Thanks to the thermoresponsive property of PNIPAm, tumor spheroids aggregated and formed at 37 °C and were readily isolated at room temperature. The matrix had suitable stiffness for spheroid culture and the formed wells were spherical and of uniform diameter throughout the PHMA, supporting the generation of uniform sized spheroids. Owing to the variabilities between cell–cell adhesion characteristics of each cell line, differences in spheroids morphology were observed. HLF generated spherical and compact spheroids while A549 displayed a more irregular morphology and took a longer time to be formed [195]. Results indicated the potential of PNIPAm-based hydrogel for reproducible, high-throughput culture of airway cells in a rapid, and cost-effective way [195, 199, 200].

#### Polydimethylsiloxane (PDMS)

Due to its strong hydrophobicity, PDMS-based compounds have been used for non-adherent cell culture. In addition to excellent biocompatibility, PDMS suits perfusion culture applications as it facilitates high oxygen permeability, which significantly enhances cellular growth and prevents hypoxia-induced necrosis, and the consequent formation of necrotic cores in 3D spheroids [201, 202]. PDMS was widely used in the development of microfluidic culture devices, which enables low cost, high-throughput culture of 3D spheroids, and efficient drug screening [203]. It was possible to fabricate tunable PDMS elastomeric wells using a one-step spontaneous interfacial reaction between aqueous droplets on liquid polydimethylsiloxane. This allowed easy and adjustable optimization of spheroid size leading to highly efficient co-culture of tumor cells and fibroblasts to replicate the in vivo complex microenvironment of cancer tissue [204]. PDMS plates developed using computer-aided design and manufacturing software showed great promise for mass production of spheroids and enhanced capacity for clinical applications. Furthermore, the fabricated plates enabled rapid formation of MSC spheroids within 24 h maintaining cell viability [205]. PDMS is a transparent, oxygen-permeable, and hydrophobic polymer that is widely used in microfluidic devices. Together with its simple preparation techniques, different surface modifications may be introduced to enhance surface hydrophobicity and facile spheroid release [20]. For SARS-CoV-2 applications, PDMS microfluidic devices showed promising results in forming 3D lung organoids that can be used to study the virus. For instance, Roy et al. proposed a new smart multichannel 3D cell culture microfluidic device using PDMS and other diverse polymeric porous/semipermeable membranes that can be implemented to form 3D lung organoids to study the pathogenesis of SARS-CoV-2 [206]. Similarly, Swank et al. developed a high-throughput microfluidic nanoimmunoassay (NIA) anti-SARS-CoV-2 antibody detection in serum or ultra-low-volume blood samples. Based on the analysis of 289 human serum samples, this method achieved a specificity of 100% and a sensitivity of 98% [207]. This device can further be used in current or future studies of serological or biomarker analysis of SARS-CoV-2.

#### Poly-$\epsilon$ caprolactone (PCL)

PCL is considered one of the easiest materials to accommodate in various types and shapes of scaffolds using different fabrication technologies. The synthetic polymer exhibits rheological and viscoelastic properties suitable for many fabrication techniques, such as a relatively low melting point (i.e., 60 °C), and solubility in several common solvents (e.g., chloroform, acetone, and dimethylformamide). Moreover, PCL is a low-cost polymer that has no isomers; hence, it has distinct biological degradation and melting points for different variants [208–210]. Electrospun nonwoven membranes of PCL and chitosan were shown to exhibit significant differences in cell proliferation depending on the fabrication technique. For instance, scaffolds fabricated by simultaneous deposition of PCL and CS fibers electrospun from separate solutions resulted in doubled proliferation rate compared to those fabricated by blending the solutions of both polymers followed by electrospinning [211]. PCL fibers were recently successfully used to culture H125 Lung adenosquamous carcinomas spheroids [212]. One major concern with synthetic polymers is the need for toxic chemicals to break bonds within the matrix, which make the release of spheroids after formation much more challenging. PCL was shown to be a potential candidate polymer in COVID-19 studies. For instance, Rezaei et al. developed 3D printed scaffold for engineering lung tissue using PCL bioink. The scaffold showed significant improvement in degradation, swelling, and mechanical characteristics. In addition, the scaffold protected the cells from apoptosis, and promoted cell adhesion, high proliferation, and cell biocompatibility in vitro [213]. In another study, Dye et al. developed adult airway epithelial cell model using PEG and PCL. In contrast to PEG that inhibited growth and maturation of cells at the immature lung progenitor stage, PLC allowed maturation of the organoids to tubelike structures, resembling the structure and diversity of cells in the adult airways [214]. These studies show the promising potential of using PLC scaffold to generate 3D lung models for SARS-CoV-2 studies (Table 2).

## Limitations of 3D cell culture models

Despite being a promising and more physiologically relevant technique, 3D culture systems showed some limitations. For example, spheroids were reported to have variable size and diameter, unequal distribution of oxygen, nutrients and paracrine factors and self-disassembly due to factors’ consumption [223–226]. In addition, 3D culture models are expensive and time and effort consuming when compared to 2D culture systems [227–229], and showed less sensitivity to treatments in drug discoveries and drug repositioning experiments [230, 231]. Despite being more biomimetic than 2D culture models, 3D models have been reported to be hard to reach to in vivo maturity [232], and some 3D models lack essential type of cells that are difficult to be involved in the culture system [232]. It is worth mentioning that each model has its own limitations based on the used cell types, ECM, and different culture conditions. For example, hydrogel models showed difficulties in culture medium change and cell harvesting [233, 234]. On the other hand, hanging droplet models are much more time and effort consuming with no ECM–cell interaction, and difficult transfer to the formed spheroids for the required analysis, in addition to heightened sensitivity to mechanical stress [235, 236]. Other limitations to 3D culture models include difficult fabrication for the ECM and the entire system, and difficult visualization due to the multicellular composition of the model [231, 236].

## Conclusions and future prospective

3D lung models are promising biomimetic models for drug screening and various in vitro studies for the current SARS-CoV-2. Despite the rapid progress in developing 3D tissue models, many technical challenges still prevent their full application in pathogen infection studies. Importantly, further studies are required to determine the best type of cells to accomplish the most physiologically relevant microenvironment for lung modeling. In addition, inadequate vasculization and precipitous core necrosis of the current 3 D lung organoids call for better techniques to maintain adequate nutrition and enduring vascularization. This can be achieved by inclusion of vascular ECs or progenitors such as PCs. Scaffold material also need further optimization, as both natural and synthetic polymers have their individual shortcomings. Natural polymers lack the characteristic mechanical properties and the required reproducibility. On the other hand, the absence of ECM components in synthetic polymers and their inertness limit their use. Integrating bioactive peptides into synthetic polymers could facilitate their use in the generation of 3D lung models. In addition, combining both natural and synthetic scaffolds in a single composite polymer represents a valid, widely applicable solution to overcome the shortcomings of each polymer individually. Studies on PDMS and HA have also shown their promising applications in generating 3D lung model and may present achievable and reliable means for better studies of lung infections and therapy. The above-mentioned techniques of 3D in vitro lung models, such as spheroids, organoids, and organ-on-a-chip can be used to better understand the underlying mechanisms of COVID-19 infection, including how the virus interacts with human cells and how it causes disease. By providing a more accurate representation of the in vivo microenvironment, these models can also be used to test the efficacy of potential antiviral drugs and to identify new drug targets. Furthermore, 3D in vitro lung models can be used to study the long-term effects of the virus on the lung tissue, which can be applied for the development of treatments for post-acute sequelae of COVID-19. They can also be used to study the effects of exposure to environmental toxins, such as air pollution, which may exacerbate the disease. Overall, 3D in vitro lung models can provide a powerful tool for understanding the underlying mechanisms of COVID-19 infection and for identifying new therapeutics to combat the disease.

## Data Availability

All data presented in this review are available and present in the text.

## Abbreviations

COVID-19: Corona virus disease of 2019
2D: Two-dimensional
3D: Three-dimensional
SARS-CoV-2: Severe acute respiratory syndrome coronavirus 2
HD: Hanging drop method
PDT: Photodynamic therapy
EGF: Epidermal growth factor
NHBEs: Normal human bronchial epithelial cells
ROCKi: Rho-associated protein kinase inhibitors
ECs: Endothelial cells
RSV: Respiratory syncytial virus
ECM: Extracellular matrix
AEC2: Type II alveolar epithelial cells
FGF7: Fibroblast growth factor 7
GSK-3β: Glycogen synthase kinase 3
TGF-β: Transforming growth factor beta
BMP4: Bone morphogenetic protein 4
AT1: Alveolar type 1
AT2: Alveolar type 2
PNECs: Pulmonary neuroendocrine cells
BASCs: Bronchioalveolar stem cells
AEC1: Alveolar epithelial type I
NGFR: Nerve-growth factor receptor
hPSCs: Human pluripotent stem cells
ACE2: Angiotensin-converting enzyme 2
TMPRSS2: Transmembrane serine protease 2
hPSC-LOs: Lung organoid model from human pluripotent stem cells
hAT2: Human alveolar stem cells
IFNs: Interferons
hBO: Human bronchial organoids
GFR: Growth factor reduced
ALI: Air–liquid interface
hiPSCs: Human-induced pluripotent stem cells
hAWOs: Human airway organoids
hALOs: Alveolar organoids
hESCs: Human embryonic stem cells
VEGF: Vascular endothelia growth factor
Dll4: Delta-like ligand 4
MMP: Matrix metalloproteinase
ECs: Endothelial cells
HIF-1: Hypoxia-inducible factor-1
ERK-1/2: Extracellular regulating kinase 1/2
MEK-1/2: Mitogen-activated protein kinase 1/2
PCs: Pericytes
HUVEC: Human umbilical vein endothelial cells
HUASC: Human umbilical artery smooth muscle cells
PDGF-B: Platelet-derived growth factor beta
DECs: Dermal endothelial cell
HDFs: Human dermal fibroblasts
MPCs: Mesodermal progenitor cells
CAM: Chicken chorion allantois membrane
EPCs: Endothelial progenitor cells
NG2: Neuron-glial antigen 2
α-SMA: Alpha-smooth muscle actin
MSCs: Mesenchymal stromal cells
bFGF: Basic fibroblast growth factor
ETV2: Erythroblast transformation-specific transcription factor 2
BM-MSCs: Bone marrow mesenchymal stem cells
GAG: Glycosaminoglycan
SF: Silk fibroin
UC-MSCs: Human umbilical cord MSCs
ASCs: Adipose stem cells
PEG: Polyethylene glycol
RGD: Arginine–glycine–aspartate–serine peptide
IGF-1: Insulin-like growth factor-1
TGF-β: Transforming growth factor beta
NGF: Nerve-growth factor
LSCs: Lung spheroid cells
RSV: Respiratory syncytial virus
HA: Hyaluronic acid
RHAMM: Receptor for hyaluronan-mediated motility
ICAM-1: Intercellular adhesion molecule 1
OCT3/4: Octamer-binding transcription factor 3/4
SOX-2: Sex-determining region Y HMG-box 2
SSEA-3: Stage-specific embryonic antigen 3
HA–CS: Hyaluronan–chitosan
HSAECs: Human small airway epithelial cells
RGD: Arginylglycylaspartic acid
RGDS: Arginine–glycine–aspartate–serine peptide
MMP-2: Matrix metalloproteinase-2
MMP-9: Matrix metalloproteinase-9
PNIPAm: Poly(N-isopropylacrylamide)
HLF: Human lung fibroblast
PDMS: Polydimethylsiloxane
PCL: Poly-$\epsilon$ caprolactone
